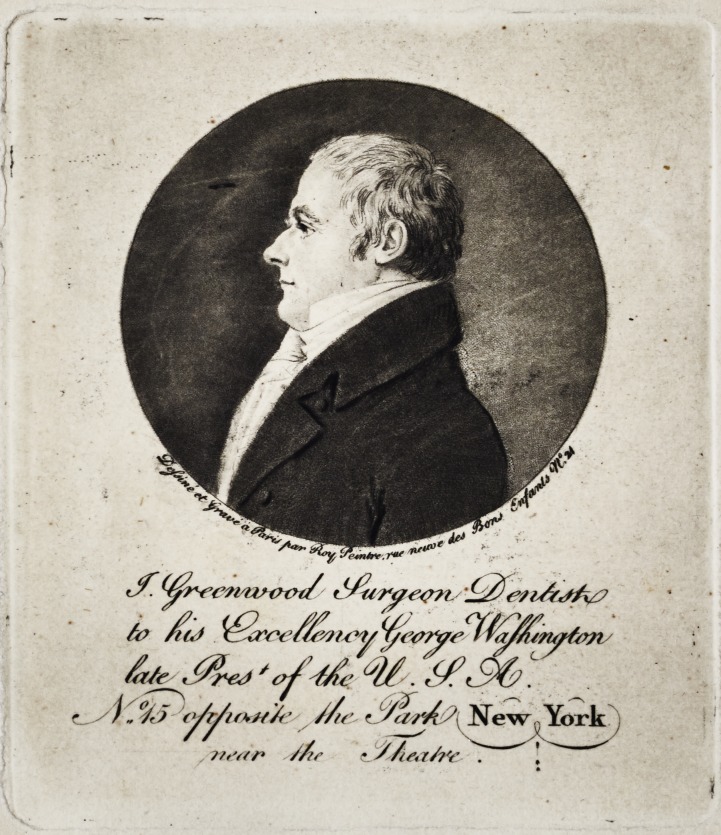# Memoirs of the Life of the Late Mr. John Greenwood

**Published:** 1839-12

**Authors:** E. Bryan


### Of the Life of the late Mr. John Greenwood,

*Mechanical and Surgeon Dentist, of New-York City: Compiled by*

E. Bryan.

The manuscript autobiography of Mr. John Greenwood, together with the notes of his son, Mr. Isaac J. Greenwood, an eminent Dentist of this city, continuing the memoirs of his father's life up to the period of his demise, having been kindly placed in my hands, at the solicitation of the Publishing Committee of this Journal, I propose to pursue the highly interesting history of the subject of these memoirs, in his own language, where it can be done with propriety, with occasional alterations in the phraseology, and where, from inaccuracy or prolixity of style, this plan shall be found to be impracticable, I shall endeavor to give a faithful abstract of all that is likely to interest, as well the general reader, as the Dental practitioner. That there should be inaccuracies of style, may be readily supposed, and will be as readily excused, when we take into account, that from a very early period of his life, such was the unsettled state of the country, growing out of the troubles existing between the Colonies and the Mother Country, that it was no easy matter to find suitable teachers, even for the most common branches of English education.

His history is, as his son justly terms it, " a plain, unvarnished tale;" but will doubtless furnish ample matter for a narrative of great interest to every American, and to every friend of liberty; and the more so, as the writer was himself an active participater in the many battles which he describes, both by sea and land, during that fearful contest for American Independence, involving the genuine principles of free government, in opposition to the mandates of arbitrary power.

The events related are without dates in the original manuscript, having been written from memory, long after they had transpired.

Mr. John Greenwood, late Mechanical and Surgeon Dentist of the city of New-York, whose name will always be memorable among his professional brethren, as having been for many years the Dentist of the great Washington, was born at Boston, Massachusetts, on the 17th of May, 1760.

His father, Isaac Greenwood, was the first practical dentist in Boston, and the only surviving son of the Rev. Isaac Greenwood, Professor of Mathematics and Natural Philosophy in Harvard College, Cambridge, Massachusetts, who emigrated from England, having been previously appointed Chaplain, on board of Admiral Montagu's Flag Ship. He was afterwards called to preside over the congregation of the old North Church, at Boston, where he appears to have been regarded as a preacher of pure christian doctrines, particularly urging upon his hearers the necessity of leading a life in accordance therewith.

The subject of these memoirs was put, to receive his education, in what was then called the North School, in Boston, where, it appears, between three and four hundred boys, the sons as well of the rich as poor, were instructed by two teachers, in merely the common rudiments of an English education, to the exclusion even of English grammar.

At the age of 13, he was taken from school and put apprentice to his uncle, Thales Greenwood, at Portland, Maine, to learn the business of a cabinet maker. Here he remained two years, when, as he says, — " the whole country was in commotion and nothing was talked of but war, liberty or death. People of all descriptions, embodying themselves into military companies, and every one that could be found that had been a soldier, or understood the manual exercise, was employed of evenings in imparting their military knowledge to the recruits. My uncle was a lieutenant of one of the companies, and as I had previously learned to play on the flute as well as on the fife, I was employed as fifer to the company." At this time the news arrived of the attack on Lexington, by the British, when fearing for the safety of his parents, young Greenwood had resolved to elope from his uncle and return to his father, in Boston, for which purpose he selected a Sunday, as the day on which he was least liable to be missed; and having put up a few articles of dress in a handkerchief, and with four and a half pistareens in his pocket, he took an early breakfast, "jumped over the back fence," and was on his way to Boston. "I set out (he says) with a light heart, sometimes running and sometimes walking, and think I must have travelled about forty miles the first day, but without feeling fatigued. I had my fife with me and my sword by my side, and was greatly caressed at the taverns where I stopt on my way, and the towns through which I passed. As the people were mustering and preparing to march towards Boston, I would take out my fife and play them a tune or two, by means of which, I travelled free of expense during the whole journey, which took me four days and a half to accomplish to Charlestown, opposite Boston, where I was detained by the sentry on duty and compelled to go to Cambridge to obtain a pass from General Ward to enable me to cross the ferry, near which, on the Boston side, was my father's residence. I obtained my pass, but still was not permitted to cross the ferry. The British Governor, Gage, gave permission to those who desired it, to leave Boston before the battle of Bunker Hill, and I now saw great crowds crossing the ferry to Charlestown, some with their furniture and some without. Charlestown was generally deserted by its inhabitants; there were, however a few houses occupied, and among them a very large tavern, which I entered, and after some conversation with the inmates they persuaded me to play them three or four tunes on my fife, at which they were very much delighted. Here I met several persons whom I knew, who induced me to go with them to Cambridge to their quarters and there strongly urged me to enlist as fifer, and after turning the matter over in my mind four or five days, during which time, I pleased myself with the idea that I might yet find an opportunity of going to Boston to see my parents; I at length yielded to their solicitation, and enlisted in Captain Bliss's company for eight months at eight dollars a month. This was late in the month of May, 1775. I was the more ready to fall in with their views when I considered my own forlorn condition; I was absent from my relatives and friends and now entirely destitute of money. We took up our quarters at the house of the Episcopal minister, who, with his family had deserted it and gone to Boston; but our quarters were far from being of an enviable description, as the house was entirely destitute of furniture; and I do not recollect that I even had a solitary blanket during our stay at this house. I remember well, that we all lay on the bare floor with our clothes on, and I recollect that I procured a stone, which I placed under my head, as a substitute for a pillow; after remaining here about two weeks, I recollected that I had a great aunt living at Andover, about twenty miles from Cambridge, whom I had a great desire to visit; I therefore procured a furlough from my captain, and entirely without the means of purchasing a meal of victuals; I nevertheless set out with a light heart soon after breakfast and had arrived within a few miles of my destination, it being late in the afternoon, when strange and unaccountable as it may be, it is nevertheless true, something seemed to prevent me from going further forward and appeared to push me back, for when I attempted to go forward I could not; but when I then turned to go back, I could travel with pleasure, very fast and without fatigue; I therefore determined to retrace my steps and travelled most of the way back, until sometime after dark, when being at length much fatigued and very hungry, having eaten nothing since breakfast, I concluded to stop at a farm house to procure something to allay the cravings of hunger and also to obtain lodging for the night. I made a good supper of a bowl of mush and milk, and wrapping myself up in a blanket, lay down on my only bed—the kitchen floor—and was presently in a sound sleep. Before day-light I was up, and without disturbing the family, proceeded on my journey to Cambridge; I had not proceeded far when I heard the firing of cannon, and quickening my pace I arrived there about ten o'clock in the morning, but was then about two miles from Bunker Hill, where the battle was raging. On my way there, I met on the road many waggons and chairs filled with the dead and the dying, and others not so badly wounded, who were enabled to walk by the assistance of others. This sight so entirely new to me, I must acknowledge, alarmed me considerably; and at that moment, I thought I would have given any thing in the world if I had not enlisted for a soldier, but my fears were quickly dispelled by witnessing the fortitude of a poor negro, who I observed had received a wound in the back of his neck, from which the blood flowed copiously. Observing that he appeared to be quite indifferent to his wound, as if free from pain, I asked him if it hurt him much?—he said no! and that as soon as it was dressed he would return to the battle. His courage seemed to have an electric effect on me, for in an instant I felt as brave as himself and never again suffered myself to be influenced by a sense of fear during the continuance of the war. At length I found the company to which I belonged, stationed on the road in sight of the battle, and in possession of two field pieces, which were posted in a position to be advantageously employed to cover a retreat in case of need. Captain Bliss was glad to see me, and complimented me for returning on so urgent an occasion before the expiration of my furlough.

The battle ended that evening by each party keeping possession of their ground. The British having met with such a warm reception, did not appear disposed to advance upon us; however, we deemed it prudent to fortify our position; accordingly we commenced next morning to build a fort on Prospect Hill, which was at the distance of about half cannon shot from their encampment. On perceiving our object, the British also set about building themselves a fort immediately opposite ours.

The night before the battle of Bunker Hill, eight hundred of our men were ordered to throw up a breast-work on Breed's Hill, which is immediately opposite Boston, and adjacent to Charlestown, and gradually slopes to the river.

Our men did not begin to work until near midnight, and then as there were but about three hundred spades and pick axes, not half the men were able to work at a time, this hasty labour effected during the night, must have been exceedingly fatiguing to our men, as those who were obliged to work were of course deprived of their accustomed rest.

This breast-work was open in the rear and commanded the approach from the river. Here all our artillery consisted of two field pieces, each three pounders.

When the battle commenced, it appeared to me like the incessant roll of a hundred drums at once. Many of our men were armed with fowling pieces instead of muskets, and of course were deficient of bayonets; and some had tomahawks instead of swords, for fighting with at close quarters; but to make up in some measure for these disadvantages, they were mostly good marksmen and also took care to load their pieces with five buckshot, besides the usual ball. They reserved their fire until the British came within pistol shot, when our men fired, and every gun brought down two or three, which caused the British troops to fall back; their lines however, were quickly filled up again and again; when they each time renewed the attack, but with a like result. At length the bayonet went to work, and many of our men who had no bayenets, used the but end of their guns, but were at last obliged to retreat, leaving the British in possession of a dear bought little piece of ground.

This hard fought battle lasted over six hours; and to give some idea of the slaughter which took place, my brother who witnessed the whole of the contest from the Boston side of the river, near the ferry, told me that he could distinctly see the British boats landing their wounded on the Boston side, and saw the men bailing out the blood and water, and fresh troops immediately took their place to return to the dreadful onslaught, and as they pushed off, he heard their wives encouraging them to join their comrades. One of the British soldiers, an Irishman, who was in the battle, was afterwards asked by another soldier, not in the battle but in Boston during the engagement, what kind of a fight they had, and what sort of fellows the Yankees were? "Faith," says he, "don't be after bothering me: sure and its myself that can tell ye all about it in a few words,—it was "*diamond cut diamond*," and that's the long and the short of it, my dear honey."

*(To be Continued.)*

## Figures and Tables

**Figure f1:**